# Serum semaphorin4C as an auxiliary diagnostic biomarker for breast cancer

**DOI:** 10.1002/ctm2.480

**Published:** 2021-08-09

**Authors:** Ya Wang, Jiahao Liu, Jiali Li, Huayi Li, Xiong Li, Long Qiao, Jie Yang, Tian Fang, Shaoqi Chen, Jingjing Ma, Junxiang Wan, Xingrui Li, Lin Zhang, Yun Xia, Yaqun Wu, Tao Xu, Jun Shao, Yaojun Feng, Ihab R. Kamel, Qifeng Yang, Zhen Li, Qinglei Gao

**Affiliations:** ^1^ Department of Gynecology and Obstetrics Tongji Hospital Tongji Medical College Huazhong University of Science and Technology Wuhan China; ^2^ Cancer Biology Research Center (Key Laboratory of the Ministry of Education) Tongji Hospital Tongji Medical College Huazhong University of Science and Technology Wuhan China; ^3^ Department of Radiology Tongji Hospital Tongji Medical College Huazhong University of Science and Technology Wuhan China; ^4^ Department of Gynecology and Obstetrics The Central Hospital of Wuhan Tongji Medical College Huazhong University of Science and Technology Wuhan China; ^5^ Department of Gynecology and Obstetrics the First Affiliated Hospital of Zhengzhou University Zhengzhou University Zhengzhou China; ^6^ Department of Obstetrics and Gynecology Wuhan Union Hospital Tongji Medical College Huazhong University of Science and Technology Wuhan China; ^7^ Leonard Davis School of Gerontology University of Southern California Los Angeles California USA; ^8^ Department of Thyroid and Breast Surgery Tongji Hospital Tongji Medical College Huazhong University of Science and Technology Wuhan China; ^9^ Department of Breast Surgery Hubei Cancer Hospital Wuhan China; ^10^ Russell H. Morgan Department of Radiology and Radiological Science The Johns Hopkins Medical Institutions Baltimore Maryland USA; ^11^ Department of Gynecology and Obstetrics Qilu Hospital of Shandong University Ji'nan China

**Keywords:** breast cancer, breast ultrasound, mammography, semaphorin4C, serum protein biomarker

Dear Editor,

Currently, breast cancer (BC) diagnosis relies on mammography guided by Breast Imaging Reporting and Data System (BI‐RADS) and biopsy when necessary.^1^ Effective blood‐based biomarkers for diagnosing BC remain insufficiently unveiled. Semaphorin4C (SEMA4C) was highly expressed in BC‐associated lymphatic endothelial cells, and soluble SEMA4C could be obtained when membrane‐bound SEMA4C was cleaved by matrix metalloproteinases.^2^ The biological significance of SEMA4C/PlexinB2 signaling in BC has been highlighted by Gurrapu et al, revealing that SEMA4C is overexpressed in BC cells, and SEMA4C/PlexinB2 signaling is essential for the growth of BC cells.^3^ Herein, we assessed serum SEMA4C as a diagnostic biomarker for BC, compared it with mammography and ultrasound, and explored the combined diagnosis of the biomarker and imaging.

We included consecutive adult women inpatients with pathologic diagnosis for breast lesions (*n *= 1833) between January 2015 and September 2019 at Tongji Hospital, Hubei Cancer Hospital, and Qilu Hospital. Patients with hepatic or renal diseases were excluded because dysfunction of the two organs could affect the clearance and excretion of serum proteins.^4^, ^5^ Detailed eligibilities are available in Supporting Information. Pathologic diagnosis, the reference standard, was performed in local centers and confirmed by the consensus of two pathologists in Tongji Hospital. Clinical data were retrospectively extracted from electronic health records by two researchers and assessed by the third investigator.

Procedures for serum SEMA4C measurements using enzyme‐linked immunosorbent assay are elaborated in Supporting Information. Serum samples were sent to Tongji Hospital for measurements, which were conducted by two researchers independently without knowledge of pathologic diagnosis and imaging assessments, and the average value was used for analysis. Machines utilized to record images and image interpretations are detailed in Supporting Information. Images were recorded by trained examiners in contributing hospitals in accordance with Mammography Quality Standards Act.^6^ Images were independently interpreted in Tongji Hospital by two radiologists with over 5 years of experience following BI‐RADS with a third radiologist as an adjudicator,^7^ being blinded to serum SEMA4C levels and pathologic diagnosis. BI‐RADS 2 and 3 breast lesions were regarded as image‐recognized benign diseases and category 4 and 5 ones as malignant.^8^

Receiver operating characteristic curve was plotted to determine the area under the curve (AUC), sensitivity, and specificity. Sensitivity, specificity, and AUC were calculated with a 95% confidence interval. The optimum cut‐off value was identified using a method depicted in other literature.^9^ Statistical analyses were performed by utilizing SPSS v23.0 (IBM Corp, NY, USA) and R v3.4.1 (www.r‐project.org), and detailed in Supporting Information. *p* values < 0.05 were regarded statistically significant.

The study design is shown in Figure 1. We included 1624 inpatients (malignant, *n *= 1027; benign, *n *= 597), and their baseline characteristics are available in Table 1. Serum SEMA4C concentration in BC (7.31 standard deviation [SD]±2.75 ng/ml) was significantly higher than that in benign breast tumors (3.54 [SD]±1.64 ng/ml). The determined optimal threshold of the biomarker for diagnosis was 5.00 ng/ml. Serum SEMA4C exhibited a higher AUC of 0.927 (95% CI 0.907–0.946) with enhanced specificity (84.8% [78.8%–89.3%]) and compromised sensitivity (83.9% [80.7%–86.7%]) to diagnose BC than mammography, which displayed an AUC of 0.788 (0.754–0.823), a specificity of 61.3% (54.5%–68.1%), and a sensitivity of 96.4% (94.9%–97.8%) (Figure 2A and Table 2). Compared with breast ultrasound, serum SEMA4C demonstrated a significantly greater AUC to identify BC (0.907 [0.891–0.922] versus 0.804 [0.783–0.825], *p *< 0.05, Figure 2B). While serum SEMA4C yielded a sensitivity of 81.8% (78.9%–84.3%) and a specificity of 83.1% (79.7%–86.0%), ultrasound demonstrated a sensitivity of 87.8% (85.6%–90.1%) and a specificity of 73.0% (69.4%–76.6%) to detect BC.

**FIGURE 1:**
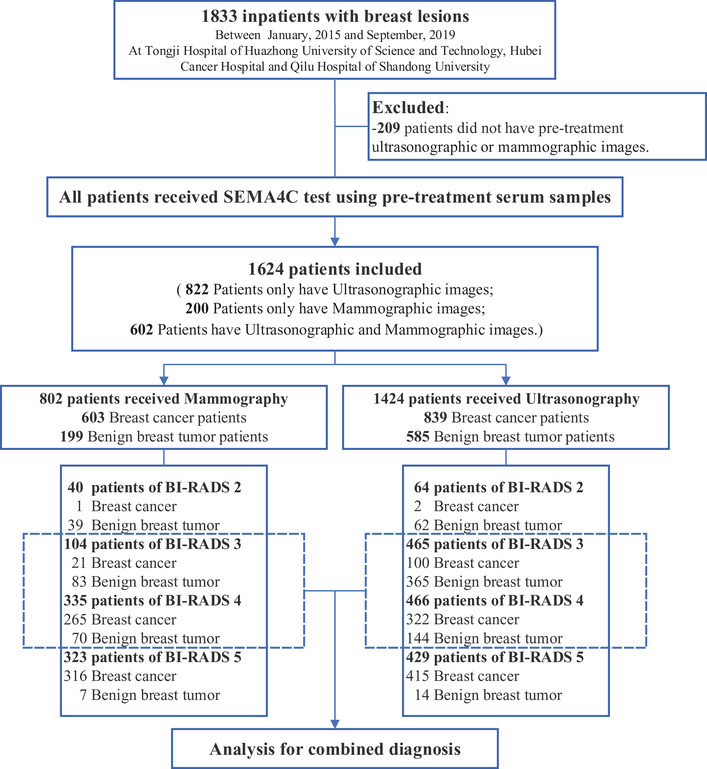
Patient enrollment flow chart. Abbreviation: BI‐RADS, breast imaging reporting and data system

**TABLE 1:** Baseline characteristics

Characteristics	Overall (*n *= 1624)	Patient with mammography (*n *= 802)	Patient with ultrasonography (*n *= 1424)
**Median age, years**	47.0 (40.0, 54.0)	48.0 (42.0, 56.0)	47.0 (40.0, 53.0)
**Age, years**			
<35	200 (12.31)	45 (5.61)	191 (13.41)
35–49	776 (47.78)	384 (47.88)	687 (48.24)
50–70	599 (36.88)	339 (42.27)	507 (35.60)
>70	39 (2.40)	28 (3.49)	32 (2.25)
Missing, *n*	10 (0.62)	6 (0.75)	7 (0.49)
**Diagnosis**			
Breast cancer	1027 (63.24)	603 (75.19)	839 (58.92)
Non‐cancer	597 (36.76)	199 (24.81)	585 (41.08)
**Breast cancer** **Histological types**			
Ductal carcinoma *in situ* (DCIS)	68 (6.62)	33 (5.47)	58 (6.91)
Invasive ductal carcinoma (IDC)	918 (89.39)	552 (91.54)	742 (88.44)
Invasive lobular carcinoma (ILC)	13 (1.27)	5 (0.83)	13 (1.55)
Missing, *n*	28 (2.73)	13 (2.16)	26 (3.10)
**Tumor Size, cm**			
<2	480 (46.74)	229 (37.98)	389 (46.36)
≥2, ≤5	421 (40.99)	274 (45.44)	338 (40.29)
<5	49 (4.77)	23 (3.81)	43 (5.13)
Missing, *n*	77 (7.50)	77 (12.77)	69 (8.22)
**Regional lymph node**			
Negative	751 (73.13)	443 (73.47)	587 (69.96)
Positive	276 (26.87)	160 (26.53)	252 (30.04)
**Metastasis**			
Negative	1023 (99.61)	602 (99.83)	835 (99.52)
Positive	4 (0.39)	1 (0.17)	4 (0.48)
**Clinical pathological subtype**			
HER2–/HR+	420 (40.90)	251 (41.63)	417 (49.70)
HER2+/HR+	318 (30.96)	181 (30.02)	191 (22.77)
HER2+/HR–	143 (13.92)	87 (14.43)	116 (13.83)
TNBC	88 (8.57)	55 (9.12)	68 (8.10)
Missing, n	58 (5.65)	29 (4.81)	47 (5.60)

Continuous variables were presented as mean and interquartile range, and categorical data were summarized as absolute frequencies and percentages.

Abbreviations: DCIS, ductal carcinoma *in situ*; HER2, human epidermal growth factor receptor 2; HR, hormonal receptor; HR+, estrogen receptor and(or) progesterone receptor positive; HR‐, estrogen receptor and progesterone receptor negative; IDC, invasive ductal carcinoma; ILC, invasive lobular carcinoma; TNBC, triple negative breast cancer.

**FIGURE 2:**
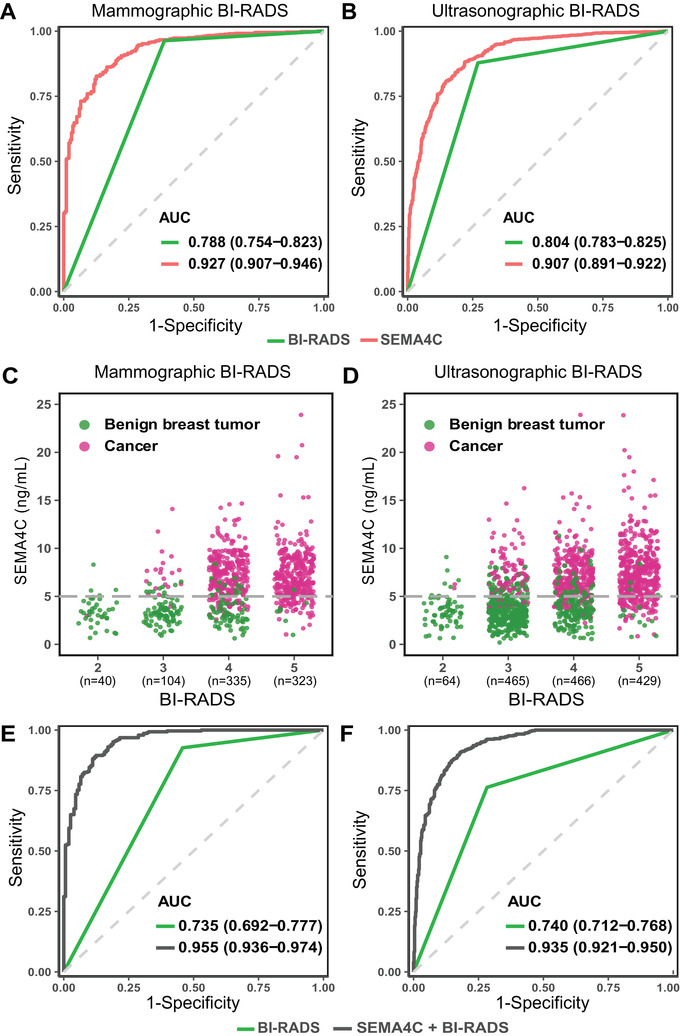
Serum SEMA4C versus imaging, serum SEMA4C in BI‐RADS 2–5 breast lesions, and combined diagnosis versus imaging in category 3 and 4 ones. (A) ROC curves for serum SEMA4C and mammography in inpatients with mammography (*n *= 802). (B) ROC curves for serum SEMA4C and ultrasound in inpatients with ultrasonography (*n *= 1424). For A and B, red curve is the ROC curve for serum SEMA4C, and green curve is the ROC curve for imaging. (C) The scatter plots of serum SEMA4C levels in mammographic BI‐RADS 2–5 breast lesions (Total, *n *= 802; category 2, *n *= 40; category 3, *n *= 104; category 4, *n *= 335; category 5, *n *= 323). (D) The scatter plots of serum SEMA4C levels in ultrasonographic BI‐RADS 2–5 breast lesions (Total, *n *= 1424; category 2, *n *= 64; category 3, *n *= 465; category 4, *n *= 466; category 5, *n *= 429). For C and D, the dotted grey horizontal line is the optimal threshold. Red dots represent breast cancer, and green dots represent benign breast tumors. (E) ROC curves for the combined diagnosis of serum SEMA4C and mammography in mammographic BI‐RADS 3/4 breast lesions (Total, *n *= 439; category 3, *n *= 104; category 4, *n *= 335). (F) ROC curves for combined diagnosis of serum SEMA4C and ultrasound in ultrasonographic BI‐RADS 3/4 breast lesions (Total, *n *= 931; category 3, *n *= 465; category 4, *n *= 466). For E and F, black curve is the ROC curve for combined diagnosis, and green curve is the ROC curve for imaging. Abbreviations: BI‐RADS, breast imaging reporting and data system; ROC, receiver operating characteristic; SEMA4C, semaphorin4C

**TABLE 2:** Imaging versus SEMA4C and combined diagnosis versus imaging in BI‐RADS 3/4 breast lesions

	Benign tumor vs. cancer	AUC (95% CI)	SN (95% CI), %	SP (95% CI), %	PPV (95% CI), %	NPV (95% CI), %
**All patients with mammography**						
Mammography	199 vs. 603	0.788 (0.754–0.823)	96.4 (94.9–97.8)	61.3 (54.5–68.1)	88.3 (85.8–90.8)	84.7 (78.8–90.6)
SEMA4C	199 vs. 603	0.927 (0.907–0.946)	83.9 (80.7–86.7)	84.8 (78.8–89.3)	94.4 (92.0–96.1)	63.3 (57.1–69.0)
**All patients with ultrasonography**						
Ultrasonography	585 vs. 839	0.804 (0.783–0.825)	87.8 (85.6–90.1)	73.0 (69.4–76.6)	82.3 (79.8–84.8)	80.7 (77.4–84.1)
SEMA4C	585 vs. 839	0.907 (0.891–0.922)	81.8 (78.9–84.3)	83.1 (79.7–86.0)	87.4 (84.8–89.6)	76.2 (72.5–79.3)
**Patients with mammographic BI‐RADS 3/4**						
Mammography	153 vs. 286	0.735 (0.692–0.777)	92.7 (89.6–95.7)	54.2 (46.4–62.1)	79.1 (74.8–83.5)	79.8 (72.1–87.5)
SEMA4C	153 vs. 286	0.937 (0.916–0.959)	86.4 (82.4–90.3)	83.7 (77.8–89.5)	90.8 (87.4–94.2)	76.6 (70.2–83.1)
Combined diagnosis	153 vs. 286	0.955 (0.936–0.974)	89.5 (86.0–93.1)	87.6 (82.4–92.8)	93.1 (90.1–96.1)	81.7 (75.8–87.6)
**Patients with ultrasonographic BI‐RADS 3/4**						
Ultrasonography	509 vs. 422	0.740 (0.712–0.768)	76.3 (72.2–80.4)	71.7 (67.8–75.6)	69.1 (64.9–73.3)	78.5 (74.8–82.2)
SEMA4C	509 vs. 422	0.907 (0.889–0.926)	82.2 (78.6–85.9)	82.9 (79.6–86.2)	80.0 (76.2–83.7)	84.9 (81.8–88.1)
Combined diagnosis	509 vs. 422	0.935 (0.921–0.950)	90.8 (88.0–93.5)	82.1 (78.8–85.5)	80.8 (77.3–84.3)	91.5 (88.9–94.0)

Abbreviations: AUC, area under the receiver operating characteristic curve; BI‐RADS, breast imaging reporting and data system; CI, confidence interval; NPV, negative predictive value; PPV, positive predictive value; SEMA4C, semaphorin4C; SN, sensitivity; SP, specificity.

The complementary diagnostic efficacy of serum SEMA4C for imaging indicated its potential as an auxiliary biomarker for BC diagnosis. Imaging could sufficiently classify BI‐RADS 2 and 5 breast lesions, but left room for improvements in category 3 and 4 ones.^10^ We thus investigated the diagnostic capabilities of serum SEMA4C in BI‐RADS 2–5 lesions (Figures 2C and 2D). For mammographic BI‐RADS 3 lesions (*n *= 104; cancer: *n *= 21), serum SEMA4C displayed an AUC of 0.967 (0.936–0.998) and a sensitivity of 95.24% (74.13%–99.75%) to diagnose BC, and resulted in only 10 false‐positives. For category 3 lesions in ultrasound (*n *= 465; cancer: *n *= 100), serum SEMA4C demonstrated an AUC of 0.940 (0.919–0.960) and a sensitivity of 88.00% (79.60%–93.37%), and the false‐positive rate was 15.34% (56/365) (Tables S1 and S2).

For mammographic category 4 lesions (*n *= 335; cancer: *n *= 265), serum SEMA4C showed an AUC of 0.922 (0.888–0.957) and a sensitivity of 85.66% (80.72%–89.53%) to detect BC, and enabled accurate classification of 78.57% (55/70) of noncancerous individuals misdiagnosed by mammography. For category 4 lesions in ultrasound (*n *= 466; cancer: *n *= 322), serum SEMA4C exhibited an AUC of 0.878 (0.842–0.914) accompanied by a sensitivity of 80.43% (75.59%–84.54%), and finely classified 78.47% (113/144) of misdiagnosed patients. Imaging could well classify BI‐RADS 2 and 5 lesions, and we did not observe the superiority of serum SEMA4C (Tables S1 and S2).

We further interrogated the united diagnosis of serum SEMA4C and imaging in category 3 and 4 lesions. The combinatorial diagnosis of serum SEMA4C and mammography displayed significantly increased AUC to diagnose BC than mammography (0.955 [0.936–0.974] versus 0.735 [0.692–0.777]; *p *< 0.05, Figure 2E). Moreover, the specificity augmented evidently from 54.2% (46.4%–62.1%) to 87.6% (82.4%–92.8%) with slightly impaired sensitivity (92.7% [89.6%–95.7%] versus 89.5% [86.0%–93.1%]; Table 2). Moreover, compared with ultrasound, the integrated diagnosis of ultrasound and serum SEMA4C resulted in improved AUC (0.935 [0.921–0.950] versus 0.740 [0.712–0.768]; *p *< 0.05, Figure 2F), sensitivity (90.8% [88.0%–93.5%] versus 76.3% [72.2%–80.4%]), and specificity (82.1% [78.8%–85.5%] versus 71.7% [67.8%–75.6%]; Table 2) to detect BC in BI‐RADS 3 or 4 lesions.

These results indicate that leveraging serum SEMA4C as an auxiliary biomarker to mammography or ultrasound in patients with BI‐RADS 3 and 4 breast lesions could potentially augment the accuracy of BC diagnosis. However, the diagnostic value of serum SEMA4C was not tested in patients with renal or hepatic diseases, in which the results should be interpreted with caution.

## CONFLICT OF INTEREST

The authors have no conflict of interest to declare.

## ETHICS APPROVAL AND CONSENT TO PARTICIPATE

The ethical committee of Tongji Hospital, Qilu Hospital, and Hubei Cancer Hospital approved all study procedures for human subjects (TJ‐C20140311). All participants provided written informed consent prior to inclusion in this study.

## AUTHORS CONTRIBUTIONS

Wang Y and Gao Q designed the study. Wang Y, Liu J, and Li J did the experiments, analyzed and interpreted the data, and wrote the manuscript. Yang J, Qiao L, and Li X run the ELISA assay. Wang Y, Liu J, Li J, Li H, and Li X contributed equally to this work. Fang T, Li H, Chen S, Ma J, Wan J, Li X, Zhang L, Xia Y, Wu Y, Shao J, and Feng Y provided, analyzed, and interpreted patients’ samples, clinical data, or both. Ihab R. Kamel and Yang Q advised on the conception and design of the study. Gao Q and Li Z conceptualized and designed the study, supervised the project, analyzed and interpreted data, and wrote the paper. All authors vouch for the respective data and analysis, approved the final version, and agreed to submit this manuscript.

## DATA AVAILABILITY STATEMENT

The data that support the findings of this study are available from the corresponding author (Gao Q) upon reasonable request.

## Supporting information

SUPPORTING INFORMATION
