# Supplementary material for: Serum semaphorin4C as an auxiliary diagnostic biomarker for breast cancer
Source: Clin Transl Med. 2021 Aug 9;11(8):e480. doi: 10.1002/ctm2.480 (PMC8351518; doi:10.1002/ctm2.480)
Supplement: Supplementary file 1 — SUPPORTING INFORMATION [file CTM2-11-e480-s001.docx]

**Supporting information**

**“Serum semaphorin4C as an auxiliary diagnostic biomarker for breast cancer”**

Wang, et al.

**Text S1.** Eligibilities

**Text S2.** Measurements of serum SEMA4C

**Text S3.** Imaging techniques and interpretation

**Text S4.** Statistical analysis

**Table S1.** Diagnostic value of serum SEMA4C in mammographic and ultrasonographic BI-RADS 2–5 breast lesions

**Table S2.** Diagnostic value of serum SEMA4C in breast lesions of different BI-RADS categories

**Text S1. Eligibilities.**

Inclusions:

1. Patients older than 18;

2. Patients with at least one mammographic or ultrasonographic assessment before initiation of any kind of biopsy or treatment (including but not limited to chemotherapy, radiotherapy, hormone therapy, and surgery);

3. Patients with a serum before initiation of any kind of biopsy or treatment (including but not limited to chemotherapy, radiotherapy, hormone therapy, and surgery);

4. Patients with histopathological diagnosis for breast lesions by surgical biopsy or mastectomy.

Exclusions:

1. Patients with known active inflammatory or autoimmune diseases;

2. Patients with known renal or liver diseases;

3. Patients with a diagnosis of other severe acute or chronic medical conditions that may interfere with the interpretation of the study results;

4. Patients with any known cancer in other internal organs or systems or a history of breast cancer ever or other cancers;

5. Pregnant or lactating women.

**Text S2.** **Measurements of serum SEMA4C**

***Chemicals for measuring SEMA4C***

Monoclonal anti-human semaphorin4C (SEMA4C) antibody (#MAB6125, R&D Systems Inc, Minneapolis, MN, US) and anti-human SEMA4C antibody (#AF6125, R&D Systems Inc) were utilized for analyses. SEMA4C recombinant protein was provided by R&D Systems Inc (#6125-S4) and was used as the standard. The biotin-labeled detection antibody was prepared with a Biotin Labelling Kit-NH2 (Dojindo Molecular Technologies Inc, Kumamoto, Japan).

***In-house SEMA4C ELISA Assay***

Serum samples were collected within 15 days before treatment, immediately separated after collection, and then stored at –80°C until analyzed. 100 μL serum sample was used for measurements. Serum SEMA4C levels were measured via a double-antibody sandwich enzyme-linked immunosorbent assay (ELISA) method using *in-house* SEMA4C detection kits. The preparation of the ELISA kit and assays was performed according to the ELISA Development Guide of R&D Systems Inc. Briefly, 96-well Nunc-Immunomicrotitre plates with MaxiSorp surface (Greiner, Germany) were coated with 100 μL of sheep anti-human SEMA4Cantibody (4 μg/ml) and incubated at 4°C overnight. The reaction was blocked with 1% bovine serum albumin. Sera were diluted to 10% and then incubated for 2 hours at 37°C. The detection antibody, biotinylated mouse anti-human SEMA4C (0.4 μg/ml), was incubated for 2 hours at 37°C, followed by the addition of 100 μL at a 1:200 dilution of streptavidin horseradish peroxidase for 20 minutes. Color development was achieved with 100 μL per well of 3,3,5,5-tetramethylbenzidine and hydrogen peroxide as a substrate, with sulfuric acid (1 mol/L) added to stop the reaction. The optical density was measured at 450 nm and referenced to 570 nm on a SpectraMax190platereader (Molecular Devices, CA, USA). The SEMA4C levels were obtained using linear regression analysis and then fitted for the standard value and multiplied by the dilution factor. When the SEMA4C level was < 0.1953 ng/mL (the lowest limit of the standard curve), the value was set to zero. Our *in-house* SEMA4C ELISA kit was optimized by evaluating the calibration curve, detection limit, recovery, cross-reactivity, dilution linearity, and day-to-day precision studies. All measurements were done in duplicate.

**Text S3.** **Imaging techniques and interpretation**

***Machines***

Mammography was performed using a full-field digital mammography unit equipped with 24 × 29 cm amorphous selenium detectors of 85-μm pixel size (Mammomat Inspiration, Siemens). Two routine views including craniocaudal and mediolateral oblique were acquired for each breast. Breast ultrasound was performed with a 7.5- to 13-MHz linear-array transducer (Volution 730, GE).

***Interpretation***

Final assessments were made in compliance with the BI-RADS reporting system,^1^ BI-RADS category 1 and 2 represented negative and benign imaging findings, respectively. Category 3 breast lesions were probably benign with > 0% but ≤ 2% likelihood of breast cancer. Category 4 and 5 breast lesions were highly suggestive of malignancy with > 2% likelihood of malignancy. As BI-RADS 2 and 3 breast lesions anticipated appropriate follow-ups, and BI-RADS 4 and 5 breast lesions potentially led to tissue biopsies, the former was regarded as image-recognized benign pathology and the latter as image-recognized malignant diseases.

**Text S4.** Statistical analysis

Receiver operating characteristic (ROC) curve was plotted to determine the area under the curve (AUC), sensitivity, and specificity. AUC, sensitivity, and specificity were calculated with a 95% confidence interval (CI). Sensitivity (specificity) means True positive (negative) rate, referring to the proportion of breast cancer patients (noncancerous individuals) who are correctly identified by serum SEMA4C or imaging as positive (negative) findings. The ROC curve was created by plotting the true positive rate (TPR) against the false positive rate (FPR) at various threshold settings. The optimum cut-off value for diagnosis was obtained by maximizing the sum of sensitivity and specificity, minimizing the overall error (square root of the sum of [1 – sensitivity]² + [1 – specificity]²), and minimizing the distance of the cut-off value to the top-left corner of the ROC curve.^2^ The median value and range of age were calculated using SPSS v23.0 (IBM Corp, NY, USA). AUC, CI, sensitivity, specificity, positive predictive value (PPV) and negative predictive value (NPV) were calculated by using the R package reportROC in R v3.4.1 (www.r-project.org). ROC curves were plotted using the R packages plotROC and ggplot2. P values < 0.05 were considered statistically significant.

Reference

1. Sickles, EA, D’Orsi CJ, Bassett LW, et al. ACR BI-RADS® Mammography. In: ACR BI-RADS® Atlas, Breast Imaging Reporting and Data System. Reston, VA, American College of Radiology; 2013.

2. Shen Q, Fan J, Yang XR, et al. Serum DKK1 as a protein biomarker for the diagnosis of hepatocellular carcinoma: a large-scale, multicentre study. *Lancet Oncol*. Aug 2012;13(8):817-26. doi:10.1016/s1470-2045(12)70233-4

**Table S1.** **Diagnostic value of serum SEMA4C in mammographic and ultrasonographic BI-RADS 2–5 breast lesions.**

|  | **BI-RADS 2** | | |  | **BI-RADS 3** | | |  | **BI-RADS 4** | | |  | **BI-RADS 5** | | |
| --- | --- | --- | --- | --- | --- | --- | --- | --- | --- | --- | --- | --- | --- | --- | --- |
| **Number of patients, *n*** | BC | Benign breast tumor | Total |  | BC | Benign breast tumor | Total |  | BC | Benign breast tumor | Total |  | BC | Benign breast tumor | Total |
| **mammography** |  |  |  |  |  |  |  |  |  |  |  |  |  |  |  |
| SEMA4C (+) | 0 | 4 | 4 |  | 20 | 10 | 30 |  | 227 | 15 | 242 |  | 259 | 1 | 260 |
| SEMA4C (–) | 1 | 35 | 36 |  | 1 | 73 | 74 |  | 38 | 55 | 93 |  | 57 | 6 | 63 |
| Total | 1 | 39 | 40 |  | 21 | 83 | 104 |  | 265 | 70 | 335 |  | 316 | 7 | 323 |
| **ultrasonography** |  |  |  |  |  |  |  |  |  |  |  |  |  |  |  |
| SEMA4C (+) | 2 | 8 | 10 |  | 88 | 56 | 144 |  | 259 | 31 | 290 |  | 337 | 4 | 341 |
| SEMA4C (–) | 0 | 54 | 54 |  | 12 | 309 | 321 |  | 63 | 113 | 176 |  | 78 | 10 | 88 |
| Total | 2 | 62 | 64 |  | 100 | 365 | 465 |  | 322 | 144 | 466 |  | 415 | 14 | 429 |

*Abbreviations*: SEMA4C: semaphorin4C. BI-RADS: Breast Imaging Reporting and Data System. BC: breast cancer.

**Table S2. Diagnostic value of serum SEMA4C in breast lesions of different BI-RADS categories**

| **BI-RADS** | **Benign tumor *vs*. cancer** | **AUC (95% CI)** | **SN (95% CI), %** | **SP (95% CI), %** | **PPV (95% CI), %** | **NPV (95% CI), %** |
| --- | --- | --- | --- | --- | --- | --- |
| **Mammography** |  |  |  |  |  |  |
| 2 | 39 *vs.* 1 | 0.641 (NA) | 0.00 (0.0–94.54) | 89.74 (74.84–96.66) | 0.0 (0.0–60.4) | 97.2 (83.8–99.9) |
| 3 | 83 *vs.* 21 | 0.967 (0.936–0.998) | 95.24 (74.13–99.75) | 87.95 (78.51–93.76) | 66.7 (47.1–82.1) | 98.6 (91.7–99.9) |
| 4 | 70 *vs.* 265 | 0.922 (0.888–0.957) | 85.66 (80.72–89.53) | 78.57 (66.83–87.13) | 93.8 (89.8–96.4) | 59.1 (48.4–69.1) |
| 5 | 7 *vs.* 316 | 0.923 (0.843–1.000) | 81.96 (77.18–85.95) | 85.71 (42.01–99.25) | 99.6 (97.5–100.0) | 9.5 (3.9–20.2) |
| **Ultrasonography** |  |  |  |  |  |  |
| 2 | 62 *vs.* 2 | 0.927 (0.862–0.993) | 100.00 (19.79–100.00) | 87.10 (75.60–93.87) | 20.0 (3.5–55.8) | 100.0 (91.7–100.0) |
| 3 | 365 *vs*. 100 | 0.940 (0.919–0.960) | 88.00 (79.60–93.37) | 84.66 (80.45–88.11) | 61.1 (52.6–69.0) | 96.3 (93.4–98.0) |
| 4 | 144 *vs.* 322 | 0.878 (0.842–0.914) | 80.43 (75.59–84.54) | 78.47 (70.69–84.70) | 89.3 (85.0–92.5) | 64.2 (56.6–71.2) |
| 5 | 14 *vs.* 415 | 0.876 (0.769–0.982) | 81.20 (77.04–84.78) | 71.43 (42.00–90.42) | 98.8 (96.8–99.6) | 11.4 (5.9–20.3) |

*Abbreviations*: BI-RADS: Breast Imaging Reporting and Data System. AUC: area under the receiver operating characteristic curve. CI: confidence interval. SN: sensitivity. SP: specificity. PPV: positive predictive value. NPV: negative predictive value. NA: not applicable.
